# Quantifying the informational value of classification images

**DOI:** 10.3758/s13428-019-01232-2

**Published:** 2019-04-01

**Authors:** Loek Brinkman, Stanny Goffin, Rens van de Schoot, Neeltje E.M. van Haren, Ron Dotsch, Henk Aarts

**Affiliations:** 1 Department of Psychology, Utrecht University, Utrecht, The Netherlands; 2 Maastricht University, Maastricht, The Netherlands; 3 Department of Methods and Statistics, Utrecht University, Utrecht, The Netherlands; 4 Optentia Research Program, Faculty of Humanities, North-West University, Potchefstroom, South Africa; 5 UMC Utrecht, Brain Centre Rudolf Magnus, Department of Psychiatry, Utrecht University, Utrecht, The Netherlands; 6 Department of Psychiatry, University Medical Center Utrecht, Utrecht & department of Child and Adolescent Psychiatry/Psychology, Erasmus Medical Centre, Rotterdam, The Netherlands

**Keywords:** Reverse correlation, Data quality, Reliability, Online experiments, Social psychology

## Abstract

Reverse correlation is an influential psychophysical paradigm that uses a participant’s responses to randomly varying images to build a classification image (CI), which is commonly interpreted as a visualization of the participant’s mental representation. It is unclear, however, how to statistically quantify the amount of signal present in CIs, which limits the interpretability of these images. In this article, we propose a novel metric, *infoVal*, which assesses informational value relative to a resampled random distribution and can be interpreted like a *z* score. In the first part, we define the infoVal metric and show, through simulations, that it adheres to typical Type I error rates under various task conditions (internal validity). In the second part, we show that the metric correlates with markers of data quality in empirical reverse-correlation data, such as the subjective recognizability, objective discriminability, and test–retest reliability of the CIs (convergent validity). In the final part, we demonstrate how the infoVal metric can be used to compare the informational value of reverse-correlation datasets, by comparing data acquired online with data acquired in a controlled lab environment. We recommend a new standard of good practice in which researchers assess the infoVal scores of reverse-correlation data in order to ensure that they do not read signal in CIs where no signal is present. The infoVal metric is implemented in the open-source rcicr R package, to facilitate its adoption.

**Electronic supplementary material:**

The online version of this article (10.3758/s13428-019-01232-2) contains supplementary material, which is available to authorized users.

*Reverse correlation* is an influential data-driven paradigm designed to uncover information used for the identification and classification of stimulus materials. The paradigm originated in the field of psychophysics and had its roots in signal-detection theory (Ahumada & Lovell, 1971; Beard & Ahumada, 1998; Eckstein & Ahumada, 2002). In recent years the paradigm has become increasingly popular, in particular in the domain of social psychology. In this field, the technique is being used to visualize that information used for social judgments—for example, in the context of gender, race, ethnicity, stereotypes, and personality traits (reviewed in Brinkman, Todorov, & Dotsch, 2017; Jack & Schyns, 2017; Sutherland, Oldmeadow, & Young, 2016; Todorov, Dotsch, Wigboldus, & Said, 2011). The obtained images are commonly regarded as visual proxies of mental representations and allow research to uncover information use and biases in social judgments that otherwise might have remained hidden. The technique may also prove valuable for clinical psychology and psychiatry, as a tool to identify aberrant mental representations and differences in information use in various mental illnesses (Brinkman et al., 2017; Richoz, Jack, Garrod, Schyns, & Caldara, 2015). One prevalent and accessible implementation of the reverse-correlation paradigm is noise-based reverse correlation (Brinkman et al., 2017; Dotsch & Todorov, 2012; Mangini & Biederman, 2004). In this implementation, stimuli are created by overlaying random noise patterns over one and the same base image (Fig. 1A; see Dotsch & Todorov, 2012; Mangini & Biederman, 2004, for details). Participants judge or classify these stimuli on some social construct—for example, trustworthiness, masculinity, or criminality (Fig. 1B). Classification images (CIs) are computed by averaging the noise patterns of the stimuli that participants indicated as being representative of the construct of interest (Fig. 1C). CIs visualize the features that are diagnostic for a given social judgment, and as such may reveal biases in mental representations. For instance, Dotsch, Wigboldus, Langner, and van Knippenberg (2008) used a reverse-correlation task to visualize what Dutch participants thought a typical Moroccan face looks like. The visualizations of Moroccan faces created on the basis of the data of more negatively prejudiced participants contained more criminal features, which was taken as evidence of a bias in these participants’ mental representation of Moroccans. The possibility that reverse correlation provides visual proxies of mental representations—which are otherwise hard to verbalize or are inaccessible—is intriguing. However, because reverse-correlation experiments always yield a CI, whether it is meaningful or based on pure random responses, the technique has a potential danger of false positives. Specifically, it is unclear how to statistically estimate whether signal is present in these images’ or whether a researcher is looking at pure noise. This is problematic, because researchers may easily trick themselves into interpreting CIs based on random data as containing a meaningful signal, essentially resulting in a Type I error.

**Fig. 1:**
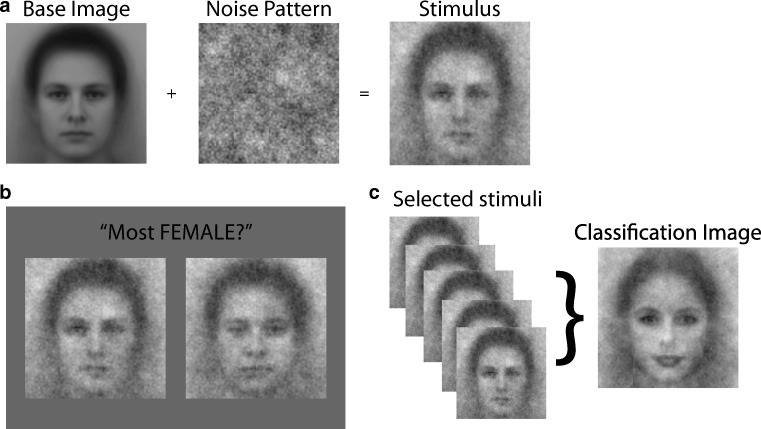
Overview of a reverse-correlation task. (A) Stimuli are generated by superimposing random noise patterns (or their inverse) on a base image (here, a morph of male and female faces). (B) In a two-image forced choice paradigm, two stimuli are presented on each trial, where the noise pattern of the stimulus on the left is the inverse of the noise pattern of the stimulus on the right. Participants choose on each trial the stimulus that best reflects the mental image that is being investigated (here, the face that is most female). (C) Individual classification images (CIs) are generated by averaging the noise patterns of the chosen stimuli and superimposing them on the base image. The individual CIs can then be averaged to obtain group-level CIs

The information contained in CIs is commonly validated subjectively—that is, by asking independent raters to judge the CIs on some construct or dimension of interest (e.g., trustworthiness or masculinity). Although these measures are the current norm (Dotsch & Todorov, 2012; Dotsch et al., 2008; Imhoff, Dotsch, Bianchi, Banse, & Wigboldus, 2011), these judgments are problematic for at least two reasons. First, they are context-sensitive. For example, CIs of male faces will be rated differently when they are embedded in CIs of female faces than when they are embedded exclusively in CIs of male faces. Second, raters may fall prey to the same problems noted above for researchers. That is, independent raters may judge CIs containing only random noise as being informative. Researchers would therefore benefit from *objective* measures to assess the amount of signal in CIs.

To date, objective metrics available to assess signal in CIs are pixel and cluster tests (Chauvin, Worsley, Schyns, Arguin, & Gosselin, 2005), which identify *where* in the CI signal is present. Although these tests are useful, they require the specification of parameters, reflecting assumptions on the strength and distribution of the signal across the pixels in the CI. Specifically, adequate filters must be chosen to smooth CIs before testing and the cluster test hinges on the specification of a threshold value that determines cluster size. Moreover, the choice of the test itself, whether a pixel or a cluster test is used, reflects an assumption on whether the underlying signal is focal or widespread, respectively.

In the present article, we introduce an easy-to-use objective metric to assess the informational value of CIs that does not require any parameters to be specified: infoVal (short for *informational value*). The infoVal metric quantifies the probability that an observed CI has not been generated by a random process and is equivalent to a modified *z* score. Because the metric solely relies on the consistencies in the data, it is robust against the aforementioned context effects. In the first section of this article, we introduce the metric and test in two simulation studies whether it adheres to typical Type I error rates rated under various task conditions (internal validity). In the second section, we test whether and how the metric correlates with markers of data quality in empirical data, such as the subjective recognizability, objective discriminability and test–retest reliability of CIs (convergent validity). In the third and final section, we showcase how infoVal can be used to compare the informational value of datasets, comparing data acquired online with data acquired in a controlled lab environment. Data, code, and materials are available on the Open Science Framework (https://osf.io/v3y5e/).

## Part I—infoVal and internal validity

The infoVal metric is based on a CI’s vector length. A vector is an object with a magnitude and a direction. In a 2-D or 3-D space, a vector can be represented as line segment or arrow, where the length of the line represents the magnitude of the vector, and the angle of the line represents its direction. In a 2-D or a 3-D space, a vector is described by two (*x*, *y*) or three (*x*, *y*, *z*) dimensions, respectively, where the vector is the line segment that starts at the origin of the graph and points toward the coordinates indicated by the values of the dimensions. By increasing the number of dimensions, a vector can be described in a multidimensional space with any number of dimensions. As such, a multidimensional vector can describe a grayscale image, such as a CI, in which each pixel of the image corresponds to a dimension. The direction of the vector describes the relative configuration of pixel intensities in the image. In the case of a CI, it might describe that the eyebrow areas are darker than the rest of the face. The CI’s vector length quantifies the extent to which ta CI deviates from the base image. The longer the vector, the more intense the changes are that are applied to the base image to get to the resulting CI. In the example above, the vector length corresponds to the extent to which the eyebrow areas are dark relative to the face. Importantly, vector length of a CI is a direct function of the similarity of the stimuli that were used to compute the CI. When participants adopt a consistent strategy for their responses and select stimuli that are more similar to each other, the resulting CI will have a longer vector length, as opposed to when responses are random. The infoVal metric quantifies the CI’s vector length relative to a reference distribution of simulated vector lengths based on random responses.

Consider a reverse-correlation task with *T* = 1,000 trials (*t* = 1, . . . , *T*), of I = 512 × 512 pixels (*i* = 1, . . . , *I*). On each trial, a participant sees two stimuli, both consisting of the base face with superimposed random noise patterns, and selects which of the two best reflects the targeted mental representation (Fig. 1B). The CI is computed by averaging the 1,000 selected noise patterns, so that the value of each pixel of the CI,${\overline{p}}_i$, is the average of the 1,000 selected stimuli for that pixel *i*. The vector length *x* of the CI is computed as the square root of the sum of squares over all pixel of the CIs, as in

1 $$ x=\sqrt{\sum \limits_{i=1}^I{\overline{p}}_i^2.} $$

**Fig. 2:**
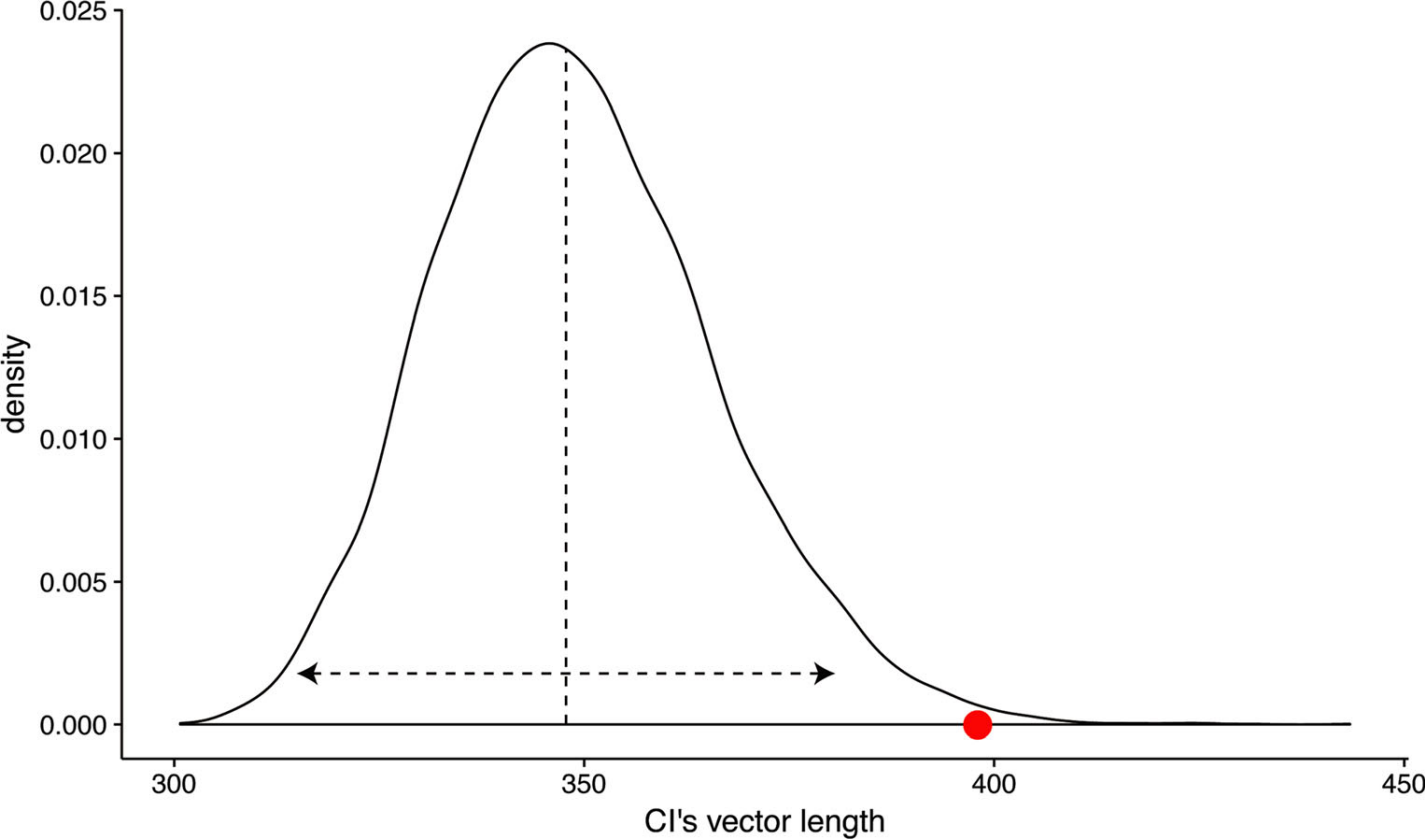
Reference distribution of vector lengths of simulated CIs based on 100% random responses (10,000 iterations, using a task with 1,000 trials with stimuli of 512 × 512 pixels). The dashed vertical line represents the median of the reference distribution, and the dashed horizontal line (with arrowheads) represents 1.96 units of the approximated standard deviation. The red dot is an example of the empirically observed CI’s vector length of 398, corresponding to an infoVal score of 3

Equation 1 is used to compute both the vector lengths of an empirically observed CI, *x*_obs_, and of simulated CIs, *x*_sim_. For *x*_obs_, the images selected by the participants are used to compute ${\overline{p}}_i$. For *x*_sim_, we simulate a participant that randomly chooses one of the two images on each trial, and these randomly selected images are used to compute ${\overline{p}}_i$ for *x*_sim_. A reference distribution of simulated vector lengths is obtained by performing 10,000 iterations of the simulation. Critically, the simulated CIs are based on the same task parameters as used in the empirical task that yielded the observed CI. This means that the simulated CIs should be based on the identical set of stimuli, including resolution, noise type, and number of stimuli. An example of such a reference distribution is depicted in Fig. 2. Note that the reference distribution is mostly normal but slightly left-skewed. The infoVal score of the observed CI is obtained by relating the vector length of the observed CI to the reference distribution of simulated vector lengths, as a modified *z* score (Iglewicz & Hoaglin, 1993). We use the modified *z* score to accommodate for the nonnormality of the reference distribution. The infoVal metric is computed as

2 $$ \mathrm{infoVal}=\frac{x_{\mathrm{obs}}-{\overset{\sim }{x}}_{\mathrm{sim}}}{\sigma_{\mathrm{sim}}}, $$

where ${\overset{\sim }{x}}_{\mathrm{sim}}$ is the *median* of the simulated vector lengths, and *σ*_sim_ is the *approximated* standard deviation of the reference distribution, computed as

3 $$ {\sigma}_{\mathrm{sim}}=k\bullet {MAD}_{{\overset{\sim }{x}}_{\mathrm{sim}}}, $$

where ${MAD}_{{\overset{\sim }{x}}_{\mathrm{sim}}}$ is the median absolute deviation of the simulated reference distribution of vector lengths, and *k* is a scaling factor with a standard value (*k* = 1.4826) for which *σ*_*sim*_ approximates the standard deviation for distributions that are close to normal (Iglewicz & Hoaglin, 1993). The vertical and horizontal dashed lines in Fig. 2 represent the median of the reference distribution and 1.96 units of the approximated standard deviation, respectively. The red dot represents an example of a vector length of an empirically observed CI with an infoVal score of 3.

### Assessment of infoVal’s internal validity through simulations

To investigate how well the infoVal metric distinguish between CIs with signal and CIs that are pure noise, we performed two simulations to establish the validity and behavior of the metric under varying circumstances. In all cases, we simulated a situation in which a participant completes a two-image forced choice (2IFC) reverse-correlation task, typical within social psychology (Brinkman et al., 2017; Dotsch & Todorov, 2012). The simulated participant’s task was to select from two random noise patterns the pattern that appeared the brightest.^1^ After doing so for a certain number of trials, a CI was constructed by using the standard approach to average all selected noise patterns, and the infoVal metric was computed as described above. In the simplest case, the simulated participant was an ideal observer who could complete the task perfectly by selecting the stimulus that had the highest average intensity value across pixels (higher intensities are brighter). Across runs, we varied the probability of random responding, *P*[random], between 0 and 1. Simulation runs in which *P*[random] equals 0 should yield identical results across runs, since the ideal observer will always make the same decisions for the same set of stimuli, providing an upper boundary for the infoVal score. This bound answers the question: What is the highest possible infoVal score if participants complete the task perfectly given the task parameters? These values should all be well above the critical value of 1.96 for an alpha of .05. The simulation runs in which *P*[random] equals 1 reflect a situation of pure noise, in which participants just select one or the other stimulus at a chance level. The CIs in the latter runs should contain no signal, because for these runs the null hypothesis of fully random responses is true. In these cases, the infoVal score should be between 0 and 1.96 in 95% of the runs. In about 5% of the runs, infoVal should be above 1.96, reflecting a Type I error rate corresponding to an alpha of .05. In Simulation 1, we simulated several other levels of *P*[random] in order to establish the general relationship between the infoVal metric and *P*[random], which can be interpreted as a combination of effect size (larger effects have smaller *P*[random]), participant motivation, and/or noise due to uncontrolled circumstances. The proportion of values that fall above the critical value of 1.96 in these cells reflects the statistical power of our metric to detect signal under these circumstances. In Simulation 2, we additionally varied the number of trials in the reverse-correlation task and the signal size (signal spanning the full image vs. some smaller part of the image). The R code for all simulations is available at http://osf.io/v3y5e.

### Simulation 1

#### Method

We simulated a 2IFC reverse-correlation task of 1,000 trials. The stimuli consisted of sinusoid noise patterns of 512 × 512 pixels generated with the R package rcicr (version 0.4.0 https://github.com/rdotsch/rcicr; Dotsch, 2017), using R version 3.3.3 (R Core Team, 2015). For a precise description of the noise, see Dotsch and Todorov (2012). Note that, as in Dotsch and Todorov’s study, on each trial two stimuli were presented, one of which was the negative of the other, to maximize visual differences. The task of the simulated participant was to choose the brightest stimulus of the two. In our first simulation, we varied the probability of random responding, *P*[random], across nine levels, ranging from 0 (ideal observer, no random responding) to 1 (chance-level responding), with equal intervals of .125. On trials with random responding, each of the two stimuli had a probability of .5 to be selected. On trials with no random responding, the stimulus with the highest average intensity value across all pixels (higher is brighter) was selected. In a single simulation run, one simulated participant completed 1,000 trials with its assigned *P*[random], after which a CI was computed according to standard procedures (Dotsch et al., 2008; Dotsch & Todorov, 2012): That is, all selected stimuli were averaged pixel by pixel. The end result was one CI per run, for which we then computed the infoVal metric as described above. Our simulation consisted of 1,000 runs for each level of *P*[random]. To compute the infoVal metric, we simulated a reference distribution of vector lengths of 10,000 CIs based on pure random responding using the same stimuli and number of trials, as we described above (Fig. 2).

#### Results

**Fig. 3:**
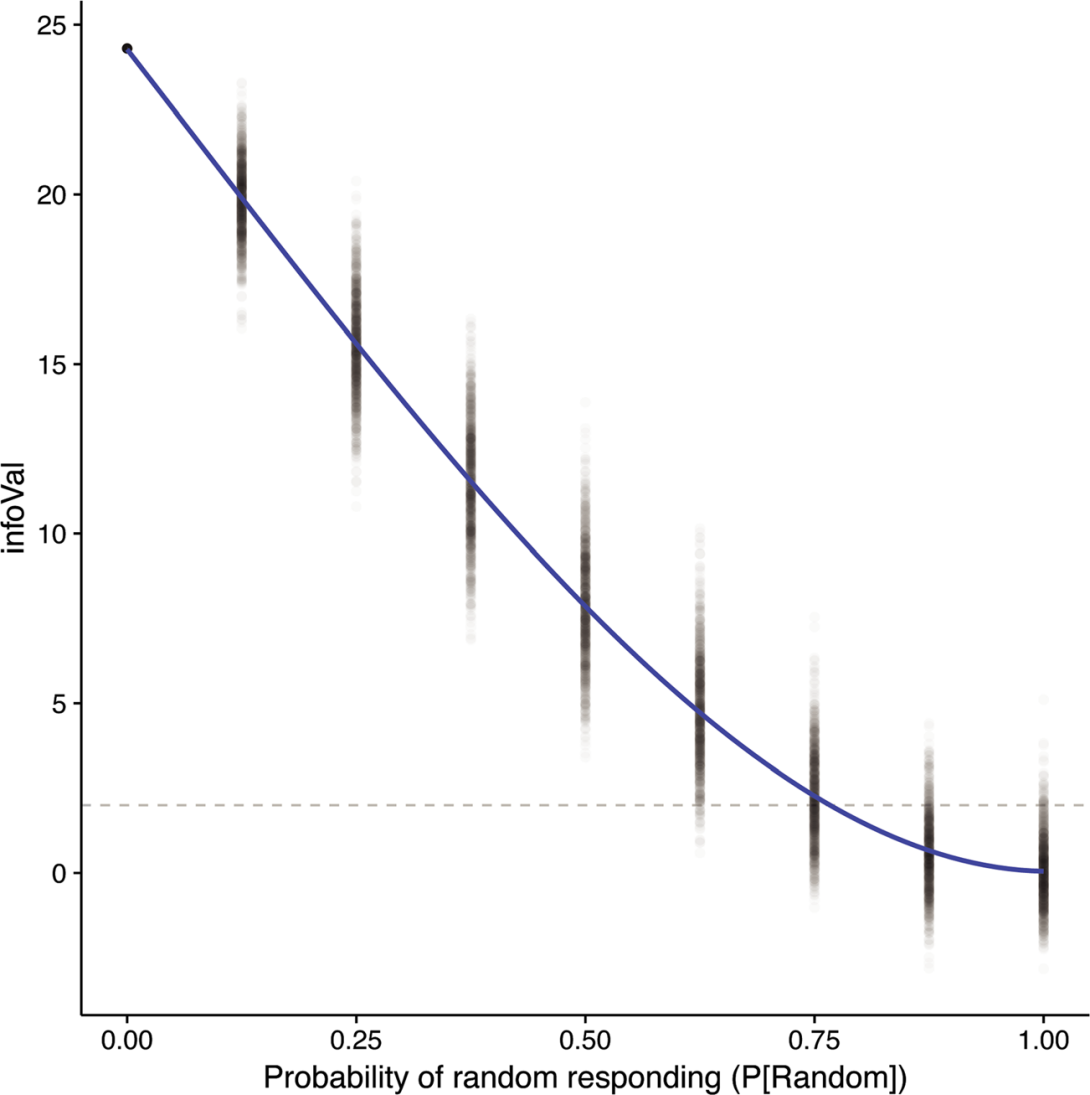
Results of Simulation 1: Relationship between random responding (*P*[Random]) and the infoVal metric. The individual runs (1,000 per level of *P*[random]) are depicted as semitransparent black points. The gray dotted line represents the critical value of infoVal = 1.96, above which the null hypothesis of random responding is rejected

Figure 3 shows the relationship between *P*[random] and the infoVal metric. The gray dotted horizontal line indicates an infoVal score of 1.96, above which the null hypothesis of random responding should be rejected. As can be seen in the figure, a greater probability of random responding leads to lower informational values (*r* = – .97, *p* < .001), and a quadratic fit explains more variance than a linear fit (*R*^2^s = .94 and .95, respectively; *p* < .001). The relationship is mostly linear, although for high levels of random responding (*P*[random] > .75), the metric is less sensitive to differences in random responding.

Importantly, the semitransparent points in Fig. 3 indicate that for 50% (or less) random responding, a decision criterion of infoVal = 1.96 has a 100% hit rate of identifying the CIs that contain signal. Type II errors (incorrectly not rejecting the null hypothesis of random responding) occur at 62.5% random responding and higher. Table 1 shows the exact false positive rates and statistical power of the infoVal metric for the various levels of random responding, separately for critical values of infoVal = 1.96 and 3. The Type I error rate is the proportion of H_0_ rejections in the last row, where *P*[random] = 1. It represents the probability to conclude that the CI was not generated by a random process, given that it actually was generated by a completely random process. Indeed, that probability is .048 for infoVal > 1.96: that is, below .05, which is the expected Type I error rate for this critical value. The Type I error rate for infoVal > 3 is .006 (where .003 would be expected for a *z* score of > 3).^2^

**Table 1:** Proportions of H_0_ rejections (H_0_ = CI generated by a random process) out of 1,000 simulation runs per level of *P*[random] for two different critical values (1.96 and 3)

*P[random]*	*infoVal > 1.96*	*infoVal > 3*
.000	1.000	1.000
.125	1.000	1.000
.250	1.000	1.000
.375	1.000	1.000
.500	1.000	1.000
.625	.975	.873
.750	.558	.286
.875	.106	.031
**1.000**	**.048**	**.006**

The bold row represents the Type I error rate, and other rows represent the power to detect signal

Moreover, the other rows in Table 1 show the probability of the correct inference that a classification was not the result of a random process. In other words, they are estimates of statistical power, given the task parameters described above (e.g., 1,000 trials, 512 × 512 pixels, and the specified noise patterns). Importantly, the metric achieves greater than 80% power for CIs that are the results of up to 62.5% random responses. This means that when, out of 1,000 trials, participants responded with signal in (at least) 375 trials, the metric can distinguish their CIs from simulated CIs based on 100% random responding with a probability well above 80%.

### Simulation 2

#### Method

In the second simulation, we explored the behavior of the infoVal metric under different task parameters and signal properties (different number of trials and different signal sizes). We distinguished between three signal sizes: When deciding between two stimuli in a reverse-correlation task, participants might pay attention (1) to all pixels (as is the case in holistic processing of the stimuli), (2) to a large number pixels (e.g., to several large features), or (3) to a small number of pixels (to some details at a very specific location—e.g., a pupil or an eyebrow). The infoVal metric may be less sensitive to a strong signal that is present only in a very small number of pixels relative to the full CI, and it might also be less sensitive to a weak signal distributed across many pixels.

To simulate these three signal sizes, we varied the number of pixels used to select the brightest stimulus. Specifically, to select the stimulus with the highest average pixel intensity, simulated participants computed the average pixel intensity of a stimulus based on a specific region: the full 512 × 512 pixel image (signal size = 100%), the 256 × 256 pixels in the middle of the image (signal size = 25%), or (3) the 32 × 32 pixels in the middle of the image (signal size = 0.39%). Orthogonally, we varied the number of trials in the reverse-correlation task (100, 300, 500, and 1,000 trials) and random responding (*P*[random] = 0, .125, .25, .5, .75, and 1.0). Every cell in the design of the simulation contained 1,000 runs. The reference distribution of vector lengths needed to compute the infoVal metric was generated using 10,000 runs separately for 100, 300, 500, and 1,000 trials, with identical stimuli, to match the task parameters in the respective cell of the simulation (four reference distributions with 10,000 samples each). The rest of the procedure was identical to that of Simulation 1.

#### Results

**Fig. 4:**
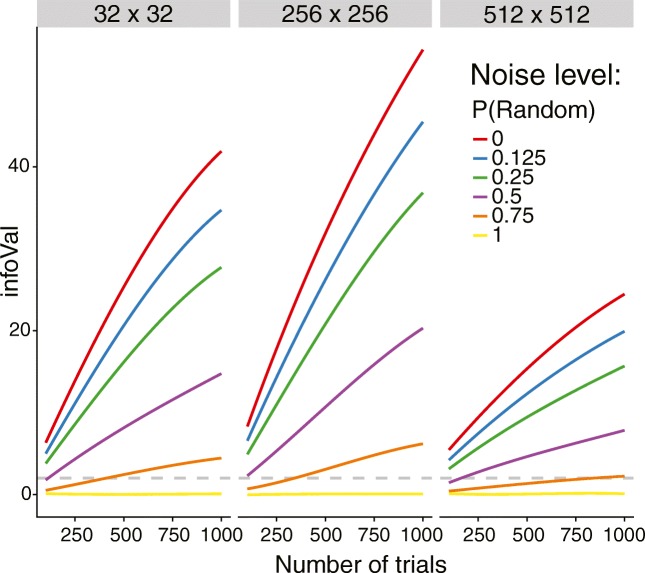
Results of Simulation 2: InfoVal scores as a function of number of trials (*x*-axis), proportion of random responding (different colored lines), and signal size (panels, from left to right: signal in 0.39%, 25%, and 100% of pixels in the stimuli)

Figure 4 shows the behavior of the infoVal metric as a function of number of trials (*x*-axis), proportion of random responding (line color), and signal size (panels). In all cases, the results of Simulation 1 are replicated: infoVal scores are higher and well above the critical value of 1.96 (gray dotted line) for lower proportions of random responding (the lower *P*[random], the stronger the signal in the CI). Moreover, we see that the metric can better differentiate between CIs with signal (low *P*[random]) and without (high *P*[random]) when more trials are used, but even with as few as 100 trials, infoVal crosses the threshold of 1.96 for CIs with up to 50% random responses. Finally, we observe that the metric is most sensitive to signal spanning 25% of the CI (256 × 256 pixels) rather than the full image (512 × 512 pixels), and loses some (but not much) sensitivity when the signal is restricted to an even smaller part of the CI (0.39%, or 32 × 32 pixels). This means that even if the signal in the CI is very small, the infoVal metric is still able to detect its presence. We can also conclude from Fig. 4 that the metric is more sensitive to a weak signal (high *P*[random]) contained in few pixels than to a strong signal (low *P*[random]) distributed across many pixels.

Table 2 shows the exact Type I error rates (in bold) and statistical power to detect signal in the CI of our metric, using infoVal > 1.96 as the criterion (see Table 3 for infoVal > 3). Importantly, irrespective of the number of trials and signal size, the proportion of infoVal scores > 1.96 when *P*[random] = 1 (no signal present) is very close to the nominal Type I error rate of alpha = .05. It can be seen from Table 3 that a stricter criterion provides better Type I error control at the cost of power to detect a signal.

**Table 2:** Proportions of H_0_ rejections (H_0_ = CI generated by a random process) out of 1,000 simulation runs, by combinations of *P*[random], signal size, and number of trials using infoVal > 1.96

*Signal size* *(pixels)*	*P[random]*	*100* *trials*	*300* *trials*	*500* *trials*	*1,000* *trials*
32	.000	1.000	1.000	1.000	1.000
32	.125	.998	1.000	1.000	1.000
32	.250	.946	1.000	1.000	1.000
32	.500	.425	.994	1.000	1.000
32	.750	.107	.326	.616	.926
**32**	**1.000**	**.044**	**.038**	**.030**	**.038**
256	.000	1.000	1.000	1.000	1.000
256	.125	1.000	1.000	1.000	1.000
256	.250	.991	1.000	1.000	1.000
256	.500	.585	.998	1.000	1.000
256	.750	.137	.402	.743	.994
**256**	**1.000**	**.032**	**.047**	**.039**	**.035**
512	.000	1.000	1.000	1.000	1.000
512	.125	.982	1.000	1.000	1.000
512	.250	.825	1.000	1.000	1.000
512	.500	.327	.785	.978	1.000
512	.750	.106	.180	.303	.564
**512**	**1.000**	**.054**	**.040**	**.048**	**.040**

Bold rows represent Type I error rates, and the other rows represent statistical power to detect signal

**Table 3:** Proportions of H_0_ rejections (H_0_ = CI generated by random process) out of 1,000 simulation runs per combination of P[random], signal size and number of trials using infoVal > 3

*Signal size* *pixels*	*P[random]*	***100*** *trials*	***300*** *trials*	***500*** *trials*	***1000*** *trials*
32	.000	1.000	1.000	1.000	1.000
32	.125	.980	1.000	1.000	1.000
32	.250	.751	1.000	1.000	1.000
32	.500	.160	.930	1.000	1.000
32	.750	.022	.128	.331	.793
**32**	**1.000**	**.003**	**.007**	**.005**	**.004**
256	.000	1.000	1.000	1.000	1.000
256	.125	.999	1.000	1.000	1.000
256	.250	.930	1.000	1.000	1.000
256	.500	.289	.982	1.000	1.000
256	.750	.025	.167	.517	.963
**256**	**1.000**	**.002**	**.007**	**.004**	**.005**
512	.000	1.000	1.000	1.000	1.000
512	.125	.879	1.000	1.000	1.000
512	.250	.548	.998	1.000	1.000
512	.500	.125	.538	.869	1.000
512	.750	.030	.055	.102	.287
**512**	**1.000**	**.008**	**.006**	**.008**	**.004**

Bold rows represent Type I error rate, other rows represent statistical power to detect signal

#### Discussion

We established the internal validity of the infoVal metric in two simulations, exploring its behavior under various parameters. The simulations showed that the metric is sensitive to changes in the amount of signal in the CI, has a satisfactory Type I error rate (close to .05 for infoVal > 1.96) and becomes more powerful when more trials are used in the task. The simulations also showed that the metric is more sensitive to signals in the CI that are constrained to a part of the image, than to signals that span the entire image, although it is sufficiently sensitive to all simulated signal sizes.

We note that the strong relation between random responding and infoVal makes it possible to decide on a critical infoVal score that corresponds with a priori stated exclusion criteria described in terms of *P*[random]. For instance, a researcher may want to include only participants who have responded randomly to at most 50% of the trials. Our work paves the way to decide on a critical value to reject the null hypothesis that a process of *P*[random] > .5 generated the CI.

Another observation is that if we assume a dependency between *P*[random] and number of trials—such that increasing the number of trials in a task also increases random responding, due to lack of participant motivation—adding more trials will not necessarily make reverse correlation more sensitive to signal (as is quantified by the infoVal metric). For instance, assuming that a signal spans 25% of the image (256 × 256 pixels), participants performing a 300-trial task might be very motivated for the first half of the task, and then after Trial 150 start responding randomly (so the total *P*[random] = .50), yielding a power of 100% to detect signal (see Table 2). If these same participants were to perform a 500-trial task, but kept on responding randomly on most of these additional trials because of the demotivation that kicks in after Trial 150, *P*[random] might be somewhere close to .75, yielding a power of around 74% to detect a true signal from the resulting CIs (see Table 2). Therefore, our simulations confirm an informal intuition of researchers, namely that it does not always pay to add more trials to a reverse-correlation task, considering that adding more trials also increases the probability of random responding due to participant demotivation.

## Part II—Convergent validity of the infoVal metric

Having established the internal validity of the infoVal metric in the previous section, we proceeded to examine its convergent validity. To this end, we computed infoVal scores for empirical data from a 2IFC task similar to the one used in the simulations in Part I. Noise patterns were constructed using the same type of noise as in Part I and were superimposed on a gender-neutral base face. Participants were instructed to select from stimulus pairs the stimuli that were either most male or female (instruction varied between participants). We tested how infoVal scores relate to three markers of CI quality: (1) A CI should readily and vividly reflect the target construct of interest (subjective recognizability), (2) a CI should objectively be more similar to CIs of the same category of target constructs, as opposed to the opposite target construct (objective discriminability), and (3) similar CIs should be obtained when the same participants repeat the task (test–retest reliability). We hypothesized that the infoVal metric should have a positive correlation with each of these markers. In addition, we explored how infoVal scores relate to response times, to verify the intuition that exceptionally fast response times correspond to CIs with little to no signal.

### Method

#### Participants

Data were collected in two samples of healthy human participants. The first sample consisted of 62 university students who were recruited at Utrecht University (The Netherlands) and performed the experiment in a controlled lab environment (“lab sample”: age = 22.3 ± 3.0 years, mean ± *SD*; 38 female, 24 male). The second sample consisted of 89 participants who were recruited using the international online participant platform Prolific (www.prolific.ac) who performed the experiment at a location of their choice (“online sample”: age = 30.4 ± 9.0 years, mean ± *SD*; 32 female, 57 male). An overview of participants’ demographics and their geographical locations can be found in the supplementary material (Fig. S1 and Tables S1 and S2).

#### Experimental design and procedure

***Session 1*** All participants performed a 2IFC reverse-correlation task on perceived gender, for which they received a monetary reward or course credits. The experiment consisted of a 2 (“sample”: online vs. lab) × 2 (“gender instruction”: male vs. female) design. Four participants in the online sample participated in multiple task conditions (e.g., in both “male” and “female” conditions). Of these participants, only the first session was included in the subsequent analyses. The task instructions and stimulus materials were identical across samples.^3^ The reverse-correlation task consisted of 1,000 trials in which participants selected from two stimuli the image that best represented their mental image of a typical male or female face (varied between participants). In the lab sample, 29 participants were in the “male” condition versus 33 in the “female” condition. In the online sample, 46 participants were in the “male” condition versus 43 in the “female” condition. The stimuli were constructed using the rcicr R package version 0.3.4.1-44 (Dotsch, 2017), which entails superimposing different random noise patterns on one and the same base image (as in the simulation studies in Part I). The base image was a greyscale image of a gender-neutral face (512 × 512 pixels), which was obtained by averaging the average male and female faces from the Karolinska face database (Lundqvist & Litton, 1998). On each trial, two stimuli were presented side by side, where the stimulus on the right side of the screen was constructed using the inverse of the noise pattern of the stimulus on the left side on the screen (see Dotsch & Todorov, 2012). Participants were instructed to use their first impression to choose the stimulus that was either most male or most female (depending on condition). Participants responded by pressing the “A” or the “L” button on their keyboard and completed the task in one go, with short breaks every 100 trials (mean time on task: 34 ± 20 min).

Participants of both lab and online samples also rated a set of CIs after completing the reverse-correlation task. These ratings focused on a different research question^4^ and are therefore not presented here. After completing the subjective ratings task, participant provided demographical information in a questionnaire.

##### Session 2

A total of 72 participants of the online sample were invited to perform the task twice (3–5 days between sessions) to assess test–retest reliability (35 were in the “male” condition and 37 were in the “female” condition). These participants were selected according to the criterion that in the first session, at least two-thirds of their response times were above 300 ms (it was not possible to perform the task at a faster pace while adhering to the instructions of the task). One of the 72 participants did not respond, and four others were excluded from the test–retest analyses because they participated in multiple task conditions (e.g., in both “male” and “female” conditions), resulting in a total sample of 67 participants for the test–retest analyses (34 in the “male” condition and 33 in the “female” condition). Directly after completing the second session of the reverse-correlation task, the participants in the retest sample also rated the 72 CIs of the first session, as a measure of subjective recognizability. The participants in the “male” condition rated the masculinity of 35 male CIs, and the participants in the “female” condition rated 37 female CIs, on a 9-point scale (for male CIs: 1 = *not masculine*, 9 = *very masculine*; for female CIs: 1 = *not feminine*, 9 = *very feminine*).

#### Data processing

##### Classification images

CIs were computed using standard procedure in R (version 3.2.3) using the rcicr package (version 0.3.4.1-44) with default settings (Dotsch, 2017). Per participant, the selected noise patterns were averaged, scaled to match the range of pixel intensities of the base image and then superimposed onto the base image to obtain an individual-level CI. Group-level CIs were computed per experimental condition, by averaging the (unscaled) average noise patterns of all participants in the respective condition, scaling the group average to match the range of pixel intensities of the base image, and then superimpose it onto the base image (auto-scaling).

#### Measures of data quality

We computed infoVal scores for all individual CIs, as we described in Part I. Subjective recognizability was quantified by average masculinity and femininity ratings of the CIs. Test–retest reliability was computed by correlating the pixel intensities of the CIs of the first and second sessions (pixel-wise correlations). Objective discriminability was operationalized as the ratio between a CI’s similarity to all other CIs within the same gender category and its similarity to the CIs within the other gender category: the objective discriminability ratio (ODR). The similarity between two CIs can be quantified by computing the Euclidean distance between the pixel intensities of the two CIs: smaller Euclidean distance equals greater similarity.^5^ The ODR is then computed as follows:

4 $$ ODR=\frac{{\overline{d}}_{across}}{{\overline{d}}_{within}}. $$

Here, ${\overline{d}}_{\mathrm{within}}$ is the average Euclidean distance of one CI to all other CIs of the same category, and ${\overline{d}}_{\mathrm{across}}$ is the average Euclidian distance of a CI to all CIs of the opposite category. In other words, we quantify the degree to which a male CI is more similar to other male CIs, then it is to female CIs (and vice versa). An ODR higher than 1 indicates that CIs can be objectively discriminated between categories.

#### Data analysis

To test the relation of the infoVal metric with the three markers of data quality we used polynomial regression, considering linear and quadratic relations.

### Results

As we described above, we computed for each participant their CI, the corresponding infoVal score, three indicators of data quality, and median response time. Here we present the results of both lab and online sample pooled together (see Part III and the supplementary material for a comparison of the online and lab data, Fig. S2).

**Fig. 5:**
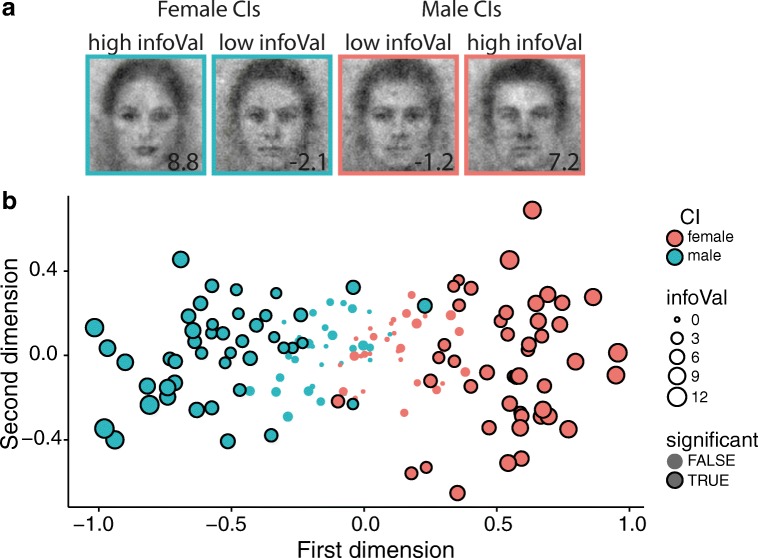
(A) Examples of the classification images (CIs) of individual participants with low and high informational value (inset in the lower-right corners of the images). CIs from the *female* and from the *male* condition are presented with a black or a red frame, respectively. (B) Multidimensional scaling plot of the individual CIs. Each dot represents a CI, and its size represents the informational value. CIs from the *female* and from the *male* condition are presented with blue and red dots, respectively. The CIs with significant amounts of informational value (infoVal > 1.96) are indicated with black outlines

**Fig. 6:**
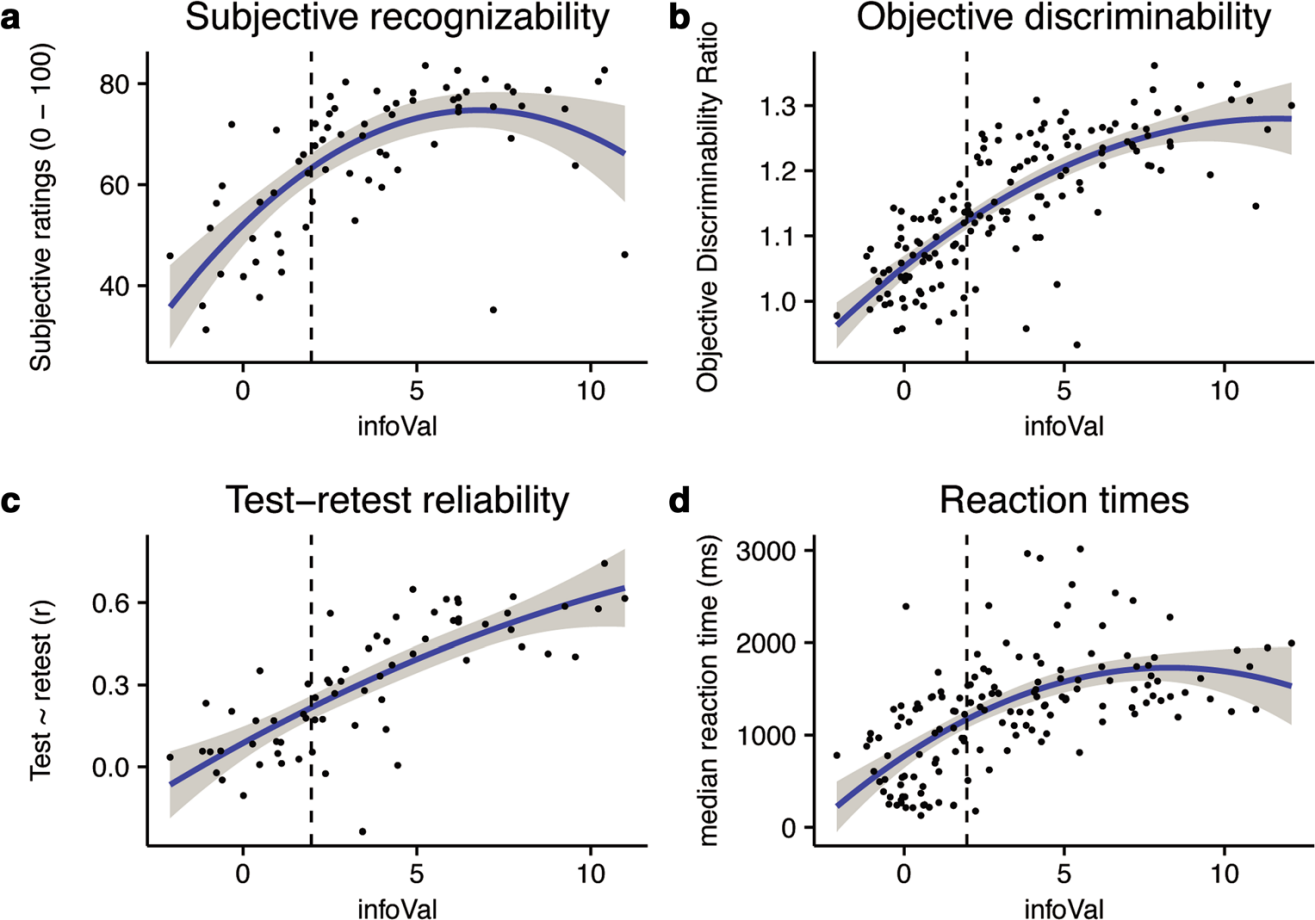
Informational value and measures of data quality. (A) Relation between infoVal scores and subjective ratings of the individual CIs by other participants. (B) Relation between infoVal and discriminability scores, computed as the ratio of the average distance *between* categories and the average distance *within* a category. (C) Relation between infoVal scores and test–retest correlations, computed as the pixel-wise correlation between the individual CIs from two sessions. (D) Relation between infoVal scores and median reaction times. In panel D, one outlier was removed for visualization purposes (median RT > 4,500 ms). This outlier was not removed for any of the statistical analyses. In all plots, dots indicate the data points of individual participants, the blue lines represent polynomial regression lines, the dashed vertical lines indicate the threshold for significant amounts of informational value (infoVal > 1.96), and the gray shading represents 95% confidence intervals

Visual inspection showed that CIs with high infoVal scores could be readily recognized as depicting male or female faces, whereas this was less clear for CIs with low infoVal scores (illustrated in Fig. 5A). This observation was confirmed by the subjective ratings of the participants, in which male and female CIs were associated more strongly with masculinity and femininity, respectively, when infoVal scores were high (Fig. 6A), *F*(2, 67) = 20.03, *p* < .001, *R*^2^ = .36. A quadratic model provided a significantly better fit than a linear model (*R*^2^s = .36 and .32, respectively; *p* < .05).

Higher infoVal scores corresponded to male and female CIs that were more objectively discriminable. This is particularly clear when the CIs are plotted in two-dimensional space (Fig. 5B) using multidimensional scaling (Jaworska & Chupetlovska-Anastasova, 2009). The scaling procedure maximizes the distance between CIs in two dimensions, based on the matrix of the Euclidean distances of all CIs. The scaling is blind to the original labels of the CIs (“male” or “female”); hence, the separation of the data in two clusters is fully data-driven. The higher the infoVal score, the more separated the male and female CIs are. Indeed, with increasing infoVal scores, the ODR of the corresponding CI increased (Fig. 6B), *F*(2, 148) = 117.80, *p* < 2.2×10^–16^, *R*^*2*^ = .61. A quadratic model provided a significantly better fit than a linear model (*R*^2^s = .61 and .58, respectively; *p* < .01).

InfoVal scores were highly correlated with test–retest reliability, where higher scores corresponded to higher test–retest reliability (Fig. 6C), *F*(2, 64) = 57.21, *p* < 1×10^–13^, *R*^2^ = .63. A quadratic model provided a significantly better fit than a linear model (*R*^2^s = .63 and .59, respectively; *p* < .001).

In addition, we explored whether and how infoVal scores related to the median reaction times of participants (Fig. 6D). The infoVal scores increased with increasing reaction times until a plateau was reached around 1,500 ms (Fig. 6D), *F*(2, 148) = 40.2, *p* < 1×10^–13^, *R*^2^ = .34. Reaction times that were extraordinarily fast (< 750 ms) were associated with low infoVal scores. A quadratic model provided a significantly better fit than a linear model (*R*^2^s = .34 and .29, respectively; *p* < .001)

### Discussion

In empirical reverse-correlation data on perceived gender, the infoVal scores shows high levels of convergent validity. The infoVal scores correlated with three indicators of data quality: CIs with high infoVal scores were subjectively perceived as more masculine or feminine, were objectively more discriminable and had higher test–retest reliability. The increases in the subjective recognizability and objective separability was most pronounced for the lower range of infoVal scores (approximately between 0 and 6), where the relation between infoVal scores and the corresponding markers of data quality was close to linear. For higher infoVal scores (approximately between 6 and 10), further increases in test–retest reliability were observed, but the increases in other markers of data quality were less pronounces (but still increased). This suggests that CIs with high infoVal scores are accurate visualization of what the participant had in mind—at least, as accurate as possible within the constraints of the task. But these higher scores do not necessarily lead to images that are regarded as more typical for the targeted mental construct. This exemplifies how infoVal scores can facilitate the interpretation of CIs: it shows whether variance in CIs across participants is due to random responding or whether it reflects the (lack of) consensus between the mental images of participants.

## Part III—Comparing data from lab and online samples using infoVal

We now turn to an application of the infoVal metric to comparing reverse-correlation datasets. More specifically, we compared data acquired via online participant platforms versus data acquired in a controlled lab environment. The use of online participant platforms has advantages over experiments performed in a lab environment. First, online data acquisition is highly efficient (i.e., cheaper, faster, and access to larger samples). Second, online pools allow testing more heterogeneous samples, improving generalizability of the findings. The drawback of online data acquisition is the lack of control on the setting in which the data is acquired. The lack of control could jeopardize data quality, as participants may be less motivated or more easily distracted. Online participant platforms have been successfully used for numerous experiments within social psychology (Peer, Brandimarte, Samat, & Acquisti, 2017), but to date have not been used for reverse-correlation experiments. We showcase the comparison of infoVal scores in reverse-correlation data acquired online versus in a controlled lab setting.

We start by comparing whether data from lab and online samples provide similar CIs and compare the infoVal scores of both samples. Finally, because participants in the lab may be more experienced with long experiments or have stronger incentives to stay motivated for a longer period of time, we compare the relationship between number of trials and infoVal scores between samples.

### Procedure and data analysis

Using the same datasets and procedures as in Part II, the group CI for “male” and “female” conditions were computed separately for the lab and online samples (using the rcicr R package, version 0.4.0 https://github.com/rdotsch/rcicr, default setting). The resulting images were compared using pixel-wise correlation. The infoVal scores of individual CIs was assessed in the online and lab samples according to the procedure described above and differences were tested using a robust independent *t* test (Mann–Whitney *U* test) to accommodate for the nonnormal distribution of the infoVal scores. Next, we investigated how the infoVal scores depended on the number of trials in both samples. We computed infoVal scores using the data of subsets of the total number of trials, for 10, 300, 500, and 1,000 cumulative trials.

### Results

**Fig. 7:**
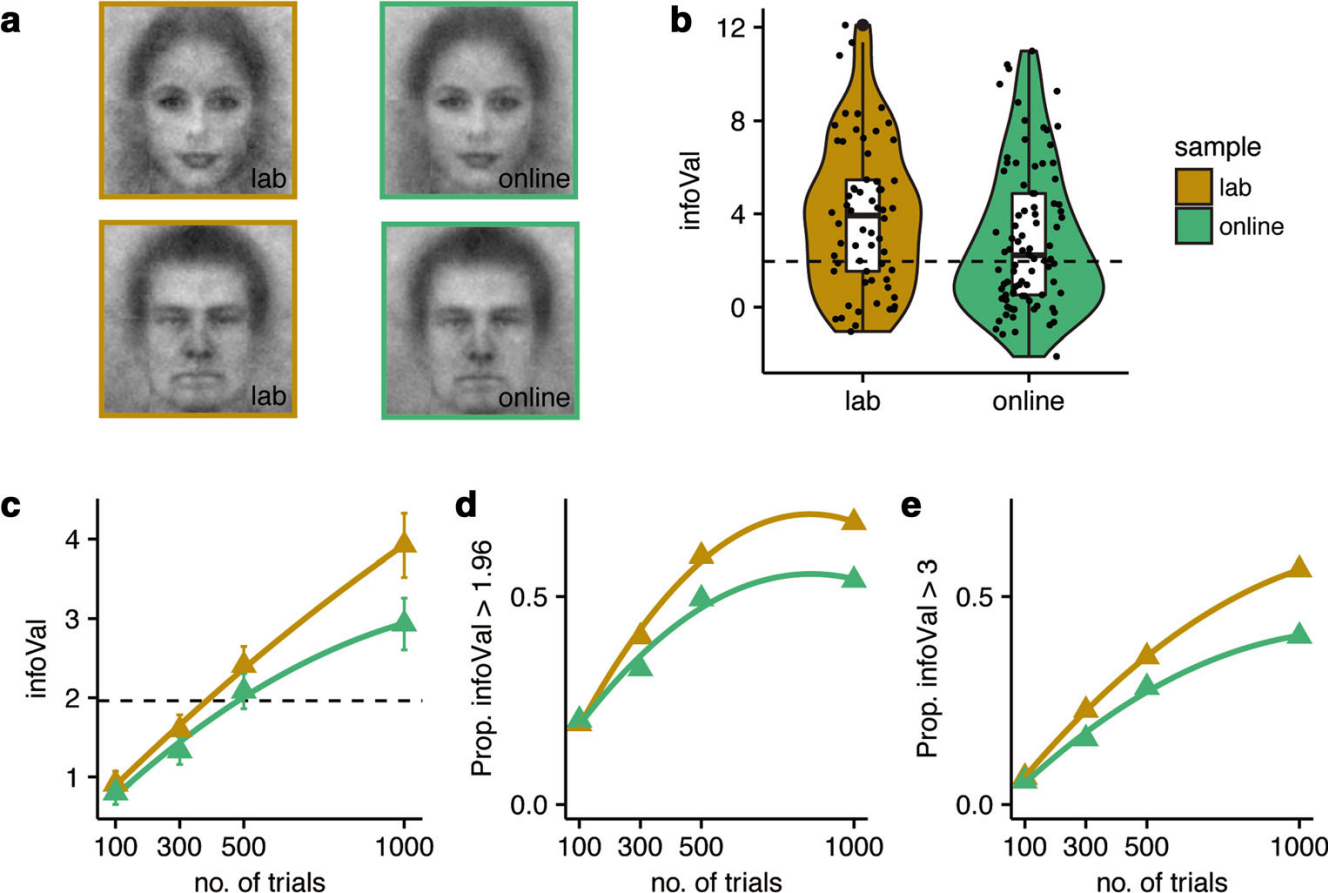
Comparison of lab and online samples. (A) Group CIs of male (bottom row) and female (top row) faces, for lab (left column) and online (right column) samples. (B) Distribution of infoVal scores for lab (orange) and online (green) samples. Black dots represent individual data points. The dashed line represents an infoVal score of 1.96. (C) Mean infoVal scores for different numbers of trials for the lab and online samples (the same conventions are followed as in panel B). Error bars represent standard errors of the means. (D + E) Proportions of infoVal scores > 1.96 (D) and > 3 (E). The same conventions are followed as in panel C

The group CIs of the online and lab samples both depict vivid images that can be readily recognized as male or female faces and are very similar across samples (Fig. 7A; male group CIs: *r* = .84, *p* < .001; female group CIs: *r* = .87, *p* < .001, pixel-wise correlations). The mean infoVal scores in the online sample were lower than those in the lab sample (Fig. 7B; online sample: 2.9 ± 0.32, lab sample: 3.9 ± 0.4, mean infoVal scores ± *SE*s; *U* = 3,290, *p* < .05, *r* = .16). Note that the ranges of infoVal scores for both the lab and online samples were large and quite similar across samples (range in lab sample: – 1 to 12; range in online sample: – 2 to 11); hence, both samples include both high- and low-quality data. The difference in mean infoVal scores is also reflected in a difference in the proportions of participants who had infoVal scores > 1.96: 68% versus 54% for the lab and online samples, respectively. In other words, to obtain the same number of high-quality CIs, one would require roughly 25% more participants when acquiring data online than when the experiment was performed in a controlled lab environment.

Moreover, we investigated for both samples how the mean infoVal scores and the proportions of CIs with infoVal scores above critical thresholds (infoVal > 1.96 or 3) were related to the number of trials (Figs. 7C–E). The relations were different for the online and lab samples. For low numbers of trials (approximately < 500 trials), both the lab and online samples show similar increases in both mean infoVal scores and the proportions of CIs with significant infoVal scores. When trial numbers are increased to 1,000 trials, both measures continue to increase, but the benefit of additional trials is less pronounced in the online sample. Also note that although the mean infoVal scores increase (nearly) linear for the whole range of trial numbers, a ceiling effect can be seen in the proportion of CIs with infoVal > 1.96. Such a ceiling effect is less pronounced for the proportion with infoVal > 3.

### Discussion

Part III provided a showcase of the application of the infoVal metric to compare the quality of reverse-correlation datasets. At the level of individual CIs, the average infoVal scores of the online sample were slightly lower than those of the lab sample. Importantly, average infoVal scores were well above the critical value of 1.96 for both samples, indicating that it is feasible to conduct reverse-correlation experiments online, albeit with a modest decrease of data quality. The feasibility of online reveres correlation experiments was further supported by the vividness and similarity of CIs obtained in lab and online samples.

Investigating the relation of infoVal scores to the number of trials lead to several interesting observations. Both the mean infoVal scores and the proportion of CIs with significant infoVal scores increased with the amount of trials. Interestingly, the benefit of adding additional trials above 500 was more pronounced for infoVal scores > 3 than for infoVal scores > 1.96. This indicates the presence of two kind of participants in the sample: motivated and less motivated participants. Motivated participant, which have infoVal scores > 1.96 after 500 trial, benefit from additional trials (500–1,000 trials), with more participants reaching infoVal scores > 3 after 1,000 trials. At the same time, less motivated participants, who have infoVal scores < 1.96 after 500 trials do not improve with additional trials (500–1,000 trials).

Moreover, increasing the number of trials is more effective in a lab setting than online, as reflected in both the mean infoVal and the proportion of CIs with significant infoVal, which indicates that adding more trials to a reverse-correlation task does not necessarily increase the sensitivity of the task. In fact, more trials may increase random responding due to demotivation, which could lead to decreased sensitivity of the task. Demotivation may come about quicker in participants of online samples, as the incentives to remain focused on the task may be less strong. When designing online reverse-correlation experiments, one should take this into consideration. It might not be worthwhile to increase trial numbers well above 500 trials. Instead, it could be more efficient to increase the number of participants in order to increase sensitivity.

## General discussion

In this article, we introduced a quantitative metric, infoVal, to quantify the amount of informational value of CIs. We showed that making decisions about signal presence based on infoVal scores adhered to expected Type I error rates in simulated reverse-correlation data (internal validity) and that the infoVal scores correlated with markers of data quality in empirical reverse-correlation data (convergent validity). In the final part, we demonstrated how infoVal scores can be applied to compare reverse-correlation datasets, comparing in this case reverse-correlation data acquired in a lab setting versus data acquired online.

The analysis of the infoVal scores in the empirical data reveals several interesting features of reverse-correlation data and inspires new practical recommendations. For instance, it shows that empirical reverse-correlation data is quite noisy. Most participants had infoVal scores in the range of 3–4. According to our simulations, this corresponds to levels of random responses of 50%–75%. By the same token, these observations indicate that the reverse-correlation method is highly sensitive to pick up even small amounts of signal in noisy data. It also shows that for numbers of trials typical in the literature (300–500 trials) large proportions of individual CIs may be unreliable. A practical recommendation is to include at least 500 trials in a reverse-correlation experiment, until better ways of power analysis are established. This is applicable for reverse-correlation experiments for perceived gender, in which the difference in mental templates (male vs. female) is large. For other social judgments, in which differences may be more subtle, the required number of trials is likely to be larger.

The implications for analysis and interpretation of CIs depend on whether researchers are interested in individual-level CIs or group CIs. Researchers who consider individual CIs can adopt the infoVal metric to set a formal criterion to exclude CIs from subsequent analysis if they fall below a certain threshold (e.g., infoVal scores < 1.96). Researchers considering group CIs can apply the infoVal metric to assess the distribution of weights of individual CIs to the group CI. In other words, it can be used to assess the proportion of participants that contributed to the group CI. These applications improve the interpretation and interpretability of current and future reverse-correlation studies. Moreover, it is important to highlight that the infoVal metric is a measure of the amount of *signal* in the data, which is not necessarily identical to the *quality* of the data. For instance, when a participant is asked to perform a reverse-correlation task on a construct for which he/she does not have a clear mental image, the resulting data will contain little signal, which in this case is an accurate description of the mental image of the participant. Therefore, when low infoVal scores are obtained, it is worthwhile to consider whether participants could have mental representations that are not well specified.

By the same token, this study shows the feasibility of online data acquisition for reverse-correlation experiments; vivid CIs were obtained for both lab and online samples that clearly represented male and female faces. The group CIs of both the lab and online samples were highly similar and readily recognizable as male or female faces. In fact, the group CIs of the online sample were at par with those of the lab sample. The proportion of individual CIs with infoVal scores > 1.96 was lower in the online sample and this difference was more pronounced as the number of trials in the task increased. The difference in infoVal scores in lab and online samples is likely because participants in the lab are more accustomed to long (and often boring) tasks. Participants in the online sample may be distracted more easily and the threshold for rushing through the experiment may be lower, as the participants remain anonymous to the researcher. For online populations in particular, designing experiment with more than 500 trials may not add substantially to the proportion of CIs with infoVal scores > 1.96. Instead, it may be more efficient to increase the number of participants.

Reverse correlation is becoming more and more popular and has many potential applications (Brinkman et al., 2017). However, to critically evaluate these and future findings and to control false positive rate in this new and emerging method, the robustness of signal in CIs needs to be assessed. The infoVal metric was developed as an easy-to-use tool to complement the few existing objective tests to assess signal in CIs, such as the pixel and cluster tests by Chauvin et al. (2005).

Although the latter test answers where in the CI signal is present by relying on assumptions about the underlying signal, infoVal scores show whether signal is present in a fully data-driven manner (no assumptions about the underlying signal). The infoVal metric quantifies the amount of signal, irrespective of whether the information is clustered. As such, the infoVal metric is also sensitive to (facial) features that are more disperse, like differences in skin color. With the introduction of the infoVal metric, we hope to have paved the way for future reverse-correlation studies to provide many more interesting findings, for which the reliability of those findings is warranted by the infoVal metric. To facilitate implementation, the metric is currently part of the developer’s version of the rcicr R package, which is freely available and has been made as user-friendly as possible.

### Conclusion

We have introduced and validated a quantitative objective metric, infoVal, to assess the amount of signal in reverse-correlation data. In addition, this study validates use of the online participant platform Prolific for reverse-correlation tasks and provides practical recommendations about obtaining reliable proxies of mental representations (CIs).

#### Author note

L.B. and R.D. designed the experiments and the infoVal metric, with valuable input form R.v.d.S. L.B. and S.G. collected and analyzed the empirical data. R.D. performed all simulations. L.B. and R.D. wrote the manuscript, with valuable input from S.G., R.v.d.S., N.v.H., and H.A. The study was supported by a Neuroscience and Cognition Utrecht, Utrecht University/UMC collaboration grant awarded to N.v.H. and H.A. R.v.d.S. was supported by a grant from the Netherlands Organization for Scientific Research (NWO), NWO-VIDI-452-14-006, and N.v.H. was also supported by a grant from the NWO, NWO-VIDI-452-11-014. We thank the students who assisted with data acquisition (Alynda Kok, Rafael Leeuwis, Valerie Kaemingk, and Amber Snelting). The data, code, and materials are available at https://osf.io/v3y5e/.

## Electronic supplementary material

ESM 1 (DOCX 230 kb)

## Footnotes

1 Although this task is more artificial than the social tasks with faces that are typically used, this should make no difference for the outcome of our simulations, but comes with the advantage of simplification.

2 Note that a higher number of simulation runs would yield a more precise estimate of the Type I error rates. We had to limit our simulation to 1,000 runs per cell in the design due to time and resource constraints. The R code has been made available for those in need of more precise estimates (https://osf.io/v3y5e/).

3 The only difference across samples was that in the lab sample the order of the stimuli was pseudo-random, whereas in the online sample the order of the stimuli was identical across participants (due to a programming error). We have no indications that this difference affected the results in any way.

4 Namely, whether CIs generated by oneself are recognized and rated higher than CIs generated by others.

5 Note that we used pixel-wise correlations to investigate similarities between the CIs, but we used Euclidean distances to investigate dissimilarities between the CIs.
