# Supplementary material for: Quantifying the informational value of classification images
Source: Behav Res Methods. 2019 Apr 1;51(5):2059–73. doi: 10.3758/s13428-019-01232-2 (PMC6797653; doi:10.3758/s13428-019-01232-2)
Supplement: Supplementary file 1 — (DOCX 230 kb) [file 13428_2019_1232_MOESM1_ESM.docx]

**Supplementary material**

**Table S1.** Demographics of lab and online participants for male and female conditions.

| *Condition* | *Lab*  *female CI* | *Lab*  *male CI* | *Online*  *female CI (session 2)* | *Online*  *male CI (session 2)* |
| --- | --- | --- | --- | --- |
| *n* | 33 | 29 | 43 (*33*) | 46 (*34*) |
| *Gender* | 19 female  14 male | 19 female  10 male | 15 (*11*) female  28 (*22*) male | 17 (*11*) female  29 (*23*) male |
| *Age ± SD* | 22.1 ± 3.1 | 22.6 ± 2.8 | 29.5 ± 9.5 (*30.9 ± 10.4*) | 31.3 ± 8.4 (*31.7 ± 9.5*) |


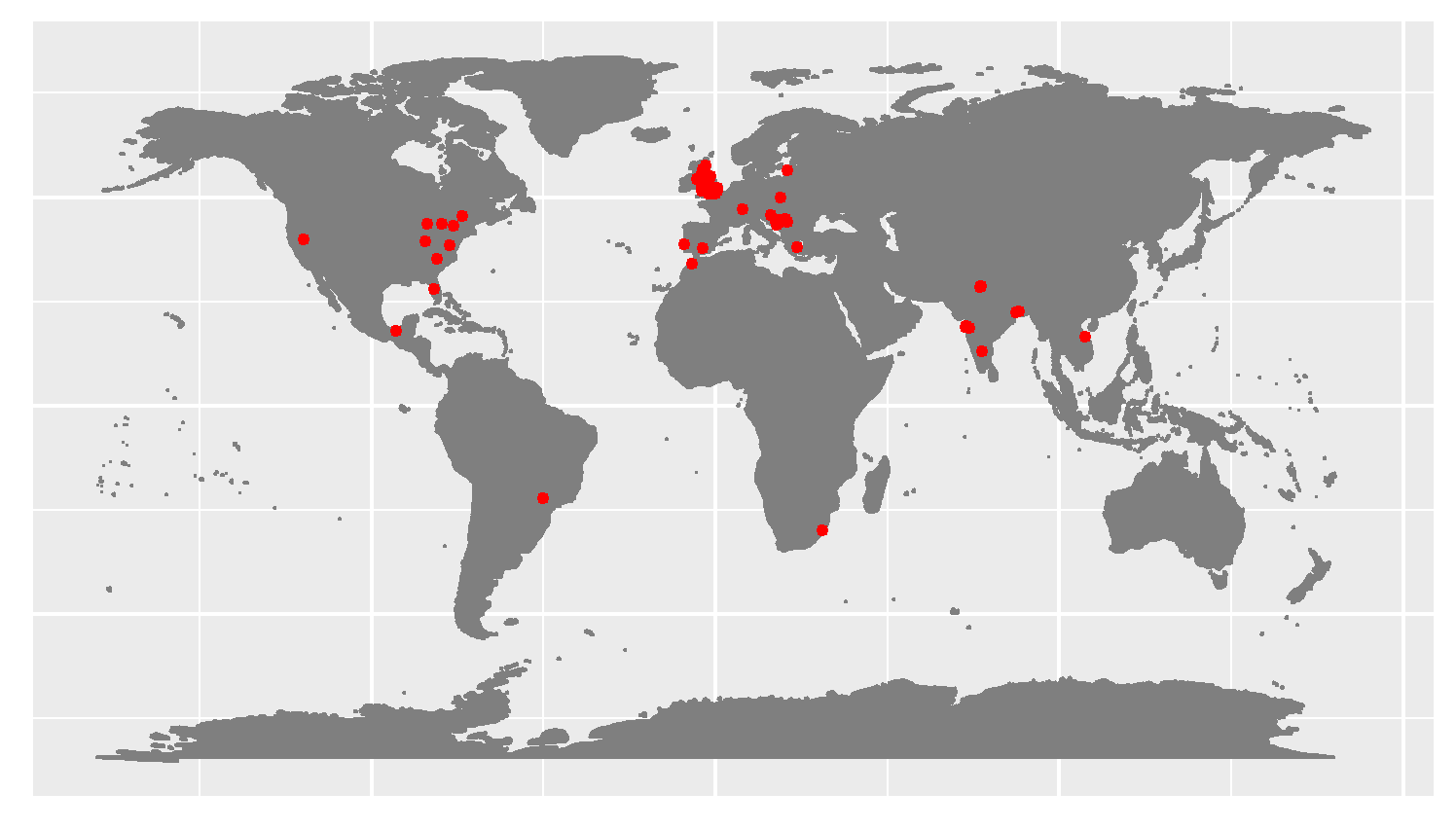
**Table S2 & Figure S1.** Overview of the countries of residence of the participants of the online sample. Red dots indicate the approximate locations of the participants while performing the task (based on IP addresses).

| *Country* | *#participants* | *Country* | *#participants* |
| --- | --- | --- | --- |
| United Kingdom | 36 | Greece | 1 |
| Bosnia and Herzegovina | 14 | Mexico | 1 |
| India | 10 | Latvia | 1 |
| Serbia | 7 | South Africa | 1 |
| United States | 7 | Vietnam | 1 |
| Canada | 2 | Portugal | 1 |
| Morocco | 2 | Croatia | 1 |
| Poland | 1 | Spain | 1 |
| Brazil | 1 | Unknown | 1 |


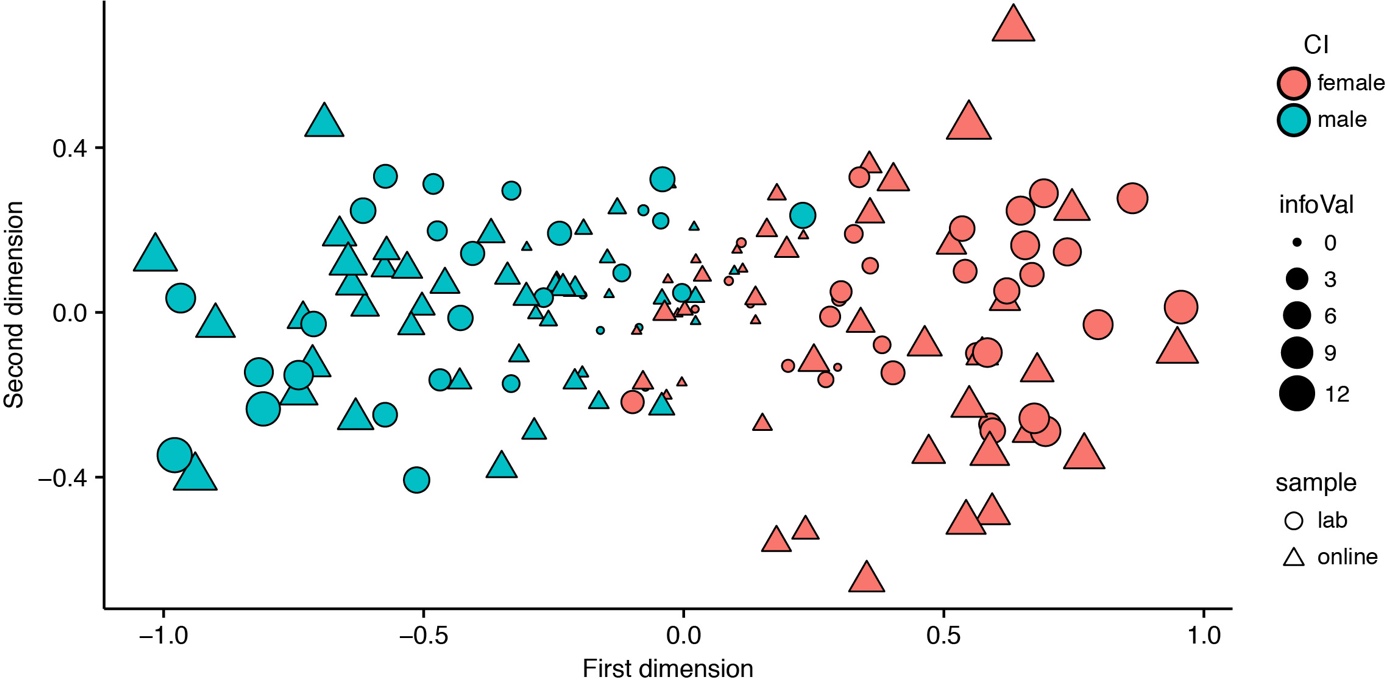


**Figure S2.**  Multidimensional scaling plot of individual classification images (CIs) for lab and online participants (comparable to Figure 2). Each circle/triangle represents a CI, and its size represents informational value. CIs from the female condition and from the male condition are presented in blue and red, respectively. CIs from lab and online participants are plotted as circles and triangles respectively.
